# Cognitive Testing of Dietary Assessment and Receipt of Nutrition Services for Use in Population-Based Surveys: Results from a Demographic and Health Surveys Pilot in Uganda^☆^

**DOI:** 10.1016/j.cdnut.2024.104518

**Published:** 2024-11-30

**Authors:** Sorrel ML Namaste, Andrea LS Bulungu, Anna W Herforth

**Affiliations:** 1 The Demographic and Health Surveys Program, ICF, Rockville, MD; 2 E3 Nutrition Lab, Brown School, Washington University in St. Louis, St. Louis, MO; 3 Department of Global Health and Population, Harvard T.H. Chan School of Public Health, Boston, MA

**Keywords:** cognitive interview, Demographic and Health Surveys, dietary assessment, indicators, nutrition services, maternal and child nutrition, minimum dietary diversity, qualitative research, survey methods, Uganda

## Abstract

**Background:**

Assessing dietary intake and receipt of nutrition services is essential for monitoring health and development, yet accurately measuring these indicators via population-based surveys remains challenging.

**Objectives:**

We investigated how respondents understood survey instructions and questions used to measure dietary intake and receipt of nutrition services. We also explored the possible influence of social desirability on responses.

**Methods:**

In a Demographic and Health Surveys pilot conducted in Uganda, women were randomly assigned to hear either a short or long introduction to dietary intake questions regarding themselves (*n* = 1116) and/or their children (*n* = 289). Dietary indicators were compared between groups using a Pearson chi-square test. Cognitive interviews about questions on both dietary intake and receipt of nutrition services were conducted with a subset of respondents with children under age 2 (*n* = 19). Transcripts were analyzed using deductive and inductive approaches.

**Results:**

Minimum dietary diversity did not differ significantly between respondents who received the short compared with long introduction to dietary questions (42.5% compared with 37.0%, respectively, for women; 39.7% compared with 46.6%, respectively, for children). The most common issues with dietary questions were inclusion or omission of items consumed in small quantities and from mixed dishes, terminology for milk feeding and yogurt consumption, and misclassification of foods by food group. Questions on receipt of nutrition services were generally well understood, although there was some confusion with terminology related to rooming-in after birth and breastfeeding observation by a health worker. Social desirability bias likely influenced responses to questions on exclusive breastfeeding in the first 2 d after birth and soda consumption and may have influenced responses to questions on meat consumption.

**Conclusions:**

Small modifications to survey questions may improve questions on dietary intake and receipt of nutrition services. Other identified issues may be best addressed through interviewer training or data interpretation. Further survey implementation research may be needed.

## Introduction

Valid data on the direct and underlying determinants of nutritional status are needed to inform and monitor programs and track global targets [1]. The need for more and better standardized data on dietary intake and receipt of nutrition services, in particular, has received increased attention [2]. In low- and middle-income countries (LMICs), these data are typically collected through multitopic population-based surveys, such as Demographic and Health Surveys (DHS) and Multiple Indicator Cluster Surveys, and other nutrition related surveys. New questions were added to the DHS model questionnaires in 2020 to incorporate recent updates to global guidance on indicators for infant and young child feeding (IYCF), minimum dietary diversity for women, consumption of sweet beverages and unhealthy foods, and receipt of nutrition services [[3], [4], [5], [6]]. However, many of these new questions, as well as many questions that have been administered for decades, have received little to no testing.

Dietary list-based recall is a method in which respondents are asked questions about drinks and foods they consumed the previous day. The questions are organized into predefined food groups and are in either a closed-ended format (i.e. respondents are asked about a discrete list of food items) or an open-ended format (i.e. respondents are asked about a food group followed with examples of items that belong in the food group). Misreporting (i.e. omission, incorrect inclusion, misclassification) has been observed in the employment of list-based recall methods [7,8]. Errors can occur with open-ended questions if the researchers’ understanding of a food group differs from the respondents’ understanding and with closed-ended questions if not all items consumed by respondents are listed in the questions. Recently adapted questions for drinks and foods used in dietary recall methods may mitigate some of these errors [9]. Using these adapted questions has yielded results similar to using a reference method in Ethiopia, Vietnam, and the Solomon Islands [10]. More information on how respondents understand terminology commonly used in dietary intake surveys is needed to help reduce or eliminate reporting errors and improve the quality of data obtained.

Regarding receipt of nutrition services, there is a limited but growing body of evidence on the performance of some of these questions. Most studies that have assessed the validity of respondent recall regarding nutrition counseling have indicated moderate to high individual and population-level accuracy [[11], [12], [13], [14], [15]]. Cognitive studies in Nepal and India identified several country-specific challenges and highlighted that respondents were more likely to recall messages on diet than on other components of nutrition counseling [16,17]. Based on the study in India, modifications were proposed to the wording of survey questions regarding the observation of breastfeeding by a health worker [16]. Further exploration is needed on the performance of survey questions regarding receipt of nutrition services and their use in different contexts.

Social desirability bias is the tendency for respondents to underreport behaviors perceived as socially objectionable and to overreport more favorable behaviors. Studies in high-income countries have found a tendency for underreporting the consumption of “unhealthy” foods and overreporting the consumption of “healthy” foods, especially among women [[18], [19], [20]]. Results of studies assessing the accuracy of reported breastfeeding practices have been mixed, but exclusive breastfeeding has been shown to be overestimated, potentially because of social desirability bias [21,22]. Research on the presence of social desirability bias and its potential impact on nutrition indicators is limited, especially in LMICs.

Standardized protocols and data collection instruments, such as DHS surveys, help to provide valid and reliable results under the assumption that respondents consistently understand the content of each survey question, can accurately recall and retrieve the information needed to answer the question, and provide the answer in the required format [23]. Misunderstandings (resulting in response errors) may occur if questions are ambiguous, provide too little information, and/or contain unfamiliar wording or jargon [24]. Cognitive interviewing allows researchers to empirically study how individuals understand, process, and respond to survey questions and can support the development of survey instruments that minimize response error.

In 2021, The DHS Program conducted a pilot study in Uganda to test how respondents understood and answered a subset of questions in the DHS model questionnaires on dietary intake and receipt of nutrition services. We tested questions that are used in the calculation of indicators on exclusive breastfeeding in the first 2 d after birth, minimum meal frequency, egg and/or flesh food consumption, zero fruit and vegetable consumption for infants and children and minimum dietary diversity, sweet beverage consumption, and unhealthy food consumption for children and women [3,4]. We also tested questions on receipt of nutrition services including indicators on maternal, infant, and child nutrition counseling, breastfeeding observation and referral, rooming-in postdelivery, and growth monitoring [5].

Here, we report quantitative and cognitive interview data from the pilot study to answer 5 research questions: *1*) How do instructions influence survey responses to dietary questions? *2*) Do respondents comprehend terminology used for certain drinks and foods? *3*) How does question formulation influence accuracy of food classification? *4*) Do respondents comprehend questions on receipt of nutrition services or actions and are they able to recall and retrieve the requested information? *5*) Does social desirability bias influence survey responses? These results can be used to modify the standard wording of survey questions on nutrition.

## Methods

This study was nested within the DHS-8 questionnaire pilot designed to test select content of the new DHS-8 standard questionnaires and modules. The Uganda Bureau of Statistics recruited a purposive sample of households from 36 clusters in peri-urban Kampala, Uganda. To be eligible for participation, households needed to include ≥1 woman aged 15 to 49 with a child under age 3, and interviews needed to be able to be conducted in either English or Luganda. All women aged 15 to 49 within the household were eligible to be interviewed. The target number of households for the overall pilot was 1000, and 1235 households were recruited between August and September 2021. A total of 1594 women aged 15 to 49 was eligible within the households.

The DHS Woman’s Questionnaire was administered to women by a female interviewer using a Computer-Assisted Personal Interviewing (CAPI) system. Informed consent was obtained prior to administering the Woman’s Questionnaire. This questionnaire covers a range of demographic, health, and nutrition topics. For the purposes of the pilot, this questionnaire was modified into a slightly shortened version of the standard and included questions that have not yet been part of the standard. For questions on intake of drinks and foods, the lists were adapted to the Uganda context based on guidance from the Global Diet Quality Project [25].

At the time households were identified as eligible, CAPI was used to automatically randomly assign women to receive either a short introduction or a long introduction to the questions on dietary intake. The purpose of the introduction was to instruct respondents to report the drinks and foods consumed “yesterday during the day or at night” (i.e. the recall period), including foods consumed as part of mixed dishes and excluding foods consumed in small amounts. The long introduction included additional cues to help respondents retrieve this information by guiding them through their day. All respondents received the same standard questions on receipt of nutrition services. Prior to pilot testing, interviewers received 2 wk of training on survey procedures and administration of questionnaires.

A subset of women who had a child under age 2 (*n* = 19) were selected through convenience sampling to participate in cognitive interviews. Selection of women was made at the time the household was selected, and a single informed consent for the survey questionnaire and the cognitive interview was also obtained at this time. The cognitive interviews were conducted immediately after the administration of the Woman’s Questionnaire using a structured interview guide (Supplemental Table 1). All respondents were asked the same set of cognitive interview questions, regardless of whether they had received a short or long introduction. Each interview was conducted by a 2-person female team comprised of the interviewer who had administered the Woman’s Questionnaire (who had received an additional week of training on conducting cognitive interviews) plus the cognitive interview trainer.

Quantitative data from the DHS pilot study were used to answer research question 1. Cognitive interviews from a subset of respondents were used to provide further information on research question 1 and answer research questions 2 to 5.

### Quantitative analysis of responses

All respondents who completed the Woman’s Questionnaire were included in the quantitative assessment of the effect of the length of the dietary intake questionnaire introduction on dietary indicators. Dietary indicators including minimum dietary diversity, egg and/or flesh food, sweet beverage, and unhealthy food consumption, as well as the proportion who had consumed drink and food groups, were calculated for the women (*n* = 1116) and (where applicable) their children under age 2 (*n* = 289). Indicators were calculated according to recommendations in FAO’s Minimum Dietary Diversity for Women guidelines and WHO-UNICEF’s IYCF indicator guidelines [3,4]. Briefly, minimum dietary diversity for women was defined as receiving foods from ≥5 of 10 predefined food groups and for children as receiving foods from ≥5 of 8 predefined food groups. These dietary indicators and drink and food groups were then compared, using a Pearson chi-square test, between respondents who had received the short introduction to the dietary questions and those who had received the long introduction. A *P* value <0.05 represented a statistically significant difference between groups.

### Cognitive interview guide

**FIGURE 1:**
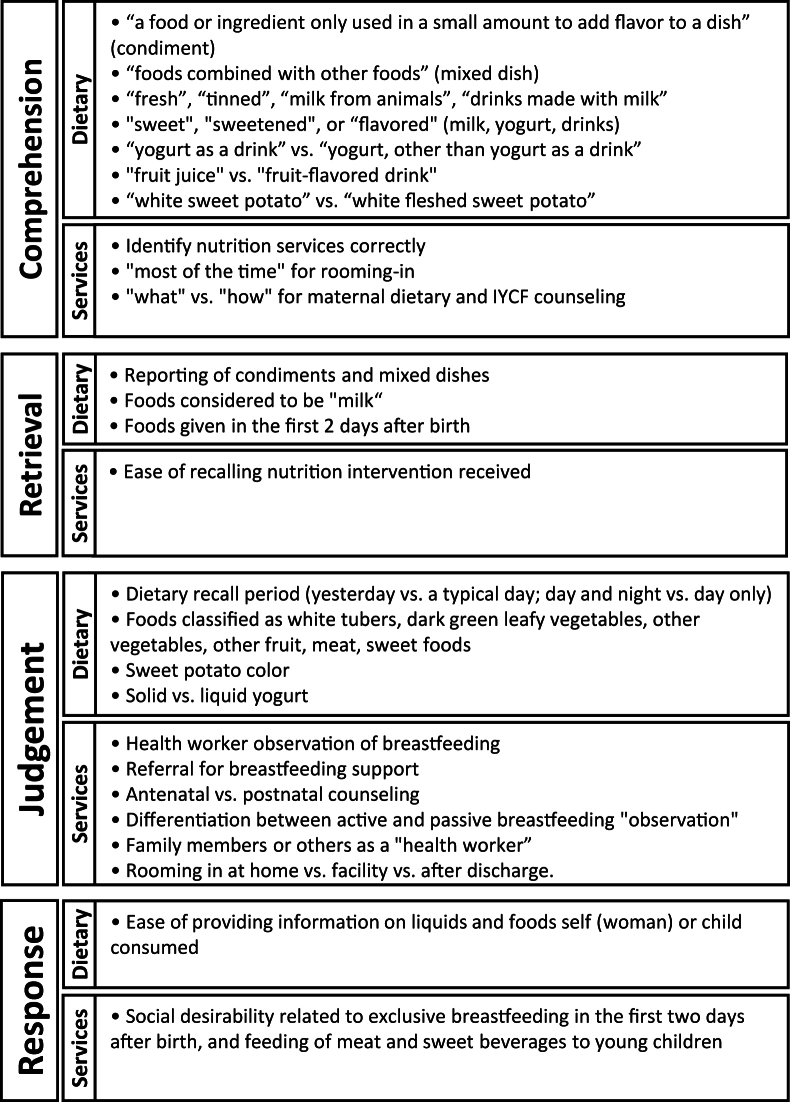
Topics covered in the structured cognitive interview guide.

The structured interview guide was developed to explore 4 key psychological processes (comprehension, retrieval, judgment, and response) related to the questions administered in the survey [23]. An iterative process was used to revise the guide during data collection to improve clarity. Specific topics investigated according to the 4 cognitive domains are presented in Figure 1.

To further explore how instructions may have influenced survey responses to dietary questions (research question 1), we probed respondents to describe the recall period in their own words, followed by questions on whether they were thinking about “yesterday” or “a typical day” when answering questions, whether they had considered items consumed at night, and how easily they had recalled their intake. We also asked respondents to describe what instructions the interviewer had given them related to mixed dishes and foods consumed in small quantities and to name representative mixed dishes and foods consumed in small quantities.

To determine whether respondents comprehended the terminology used for certain drinks and foods (research question 2), we asked them what they thought certain terms meant and to name drinks and foods they associated with each term. For milk, we were interested in whether “fresh” would be interpreted as pasteurized and raw, “tinned” would be interpreted to include items like sweetened condensed milk or nondairy products (which do not belong in this drink group), whether other milk-based drinks that do belong in this group (e.g. milk-based smoothies) would be considered as “milk,” and whether using the term “milk from animals” would help clarify that breastmilk or infant formula did not belong in the milk group. Given the development of a new sweet beverage indicator [4], we explored understanding of the terms “sweetened,” “sweet,” and “flavored” in reference to different kinds of drinks. We also tested whether respondents could distinguish between yogurt drinks and solid yogurt. This was necessary because only liquid yogurt counts toward the sweet beverage indicator and because only solid yogurt should be considered as a response to a question on the frequency of feeding solid, semisolid, or soft foods, which counts toward the calculation of the minimum meal frequency indicator. Lastly, we asked respondents whether they understood the term “instant noodles” and which of 2 terms—“orange or yellow inside” or “orange- or yellow-fleshed”—would be preferable for identifying vitamin A-rich sweet potatoes.

To assess whether question formulation influenced accuracy of food classification (research question 3), we sought to understand the potential for misclassification in both open-ended and closed-ended questions related to dietary intake. We asked respondents to name representative foods in each food group and asked additional questions related to a few specific foods known to be problematic (e.g. “Is cabbage a dark leafy green vegetable or another vegetable?”). To determine if respondents understood words commonly used to describe the colors of sweet potatoes as intended and whether a visual aid would help respondents accurately classify sweet potatoes, we showed them 4 photographs of sweet potatoes purchased in Kampala markets—*1*) white non-biofortified, *2*) yellow non-biofortified, *3*) vitamin A-biofortified but light orange in color, and *4*) vitamin A-biofortified and dark orange in color—and asked them to name the color (Supplemental Figure 1).

To evaluate whether respondents comprehended questions on receipt of nutrition services (research question 4), they were asked to describe each service and their ease of recalling it. Respondents who had received dietary counseling were additionally asked to summarize the content provided. For the question about observation of breastfeeding, we explored whether respondents understood that “observation” refers to observation by a health worker (not by family members or others). We also tested whether adding the word “correctly” to the question would reduce ambiguity (e.g. “Observe (NAME) breastfeeding?” compared with “Observe (NAME) breastfeeding correctly?”). Finally, for the question on rooming-in postdelivery, we explored whether respondents had a common understanding of the concept of the mother and newborn staying together “most of the time” and whether they considered both the time they were in the facility and the time after they had returned home.

Lastly, in determining whether social desirability influenced survey responses (research question 5), we focused on survey questions about exclusive breastfeeding in the first 2 d and feeding of soda and meat to young children. We asked respondents generally about norms in the community and whether they would or thought others would answer these questions honestly.

### Analysis of cognitive interviews

The cognitive interviews were both audio-recorded and documented through field notes, translated from Luganda as needed, and transcribed verbatim. All transcripts were checked for accuracy and completeness and were analyzed using both deductive and inductive approaches [16,[26], [27], [28]].

The cognitive interview data were first tabulated by research question using Microsoft Word, analyzed for common and unique patterns, and categorized into key findings. For each of the 5 research questions, we looked for patterns in relevant survey responses according to whether respondents had received the short or long introduction. We also looked for discrepancies between survey responses in relation to the information provided in the cognitive interview.

Second, to identify emergent themes, the transcripts were imported into NVivo 12 and coded using an a priori codebook based on the research questions and structured interview guide. Themes were identified from the coded data, and the codebook was revised as new themes emerged during analysis. The qualitative analysis was conducted by 1 researcher (AB); the results of the qualitative analysis were reviewed by 2 researchers (AH, SN). The researchers discussed findings and resolved any differences in interpretation.

### Ethical approval

The study was approved by the ICF Institutional Review Board, Makerere School of Health Sciences Research Ethics Committee (MAKSHSREC-2021-106), and the Uganda National Council for Science and Technology (HS1529ES). All study participants provided informed consent before administering the questionnaire and cognitive interviews.

## Results

A total of 1116 women completed the dietary data questions for women in the Woman’s Questionnaire, of whom 548 received a short introduction to the dietary questions for themselves and, where applicable, their children (*n* = 156), and 568 received the long introduction for themselves and, where applicable, their children (*n* = 133). The numbers of respondents in the comparative groups were not exactly equal, as randomization had been performed at the household rather than questionnaire level. No significant differences were found in the characteristics of respondents who had received the short introduction and the characteristics of those who had received the long introduction (Table 1). The Woman’s Questionnaire took a mean of 31 min (range: 3–276 min) to administer (based on available data from 1051 questionnaires).

**TABLE 1:** Respondent characteristics.

Characteristic	Dietary introduction Women 15–49 y^1^		Dietary introduction Children 6–23 mo^2^		Cognitive interview^3^
	Short *N* = 548	Long *N* = 568	Short *N* = 156	Long *N* = 133	*N* = 19
Woman’s age, y^4^	26.6 (0.3)	26.8 (0.3)	26.5 (0.5)	26.8 (0.5)	25 (19, 42)
Language, *n* (%)					
Luganda	465 (84.9)	461 (81.2)	129 (82.7)	102 (76.7)	8 (47.1)
English	27 (4.9)	29 (5.1)	9 (5.8)	9 (6.8)	2 (11.8)
Other	56 (10.2)	78 (13.7)	18 (11.5)	22 (16.5)	7 (41.2)
Marital status, *n* (%)					
Married/living together	367 (68.2)	359 (64.3)	137 (91.3)	112 (91.1)	16 (94.1)
Divorced/separated	46 (8.6)	59 (10.6)	11 (7.3)	8 (6.5)	1 (5.9)
Widowed	6 (1.1)	7 (1.3)	0 (0)	0 (0)	0 (0)
Never married	119 (22.1)	133 (23.8)	2 (1.3)	3 (2.4)	0 (0)
Education, n (%)					
No education	16 (2.9)	18 (3.2)	1 (<1.0)	2 (1.5)	1 (5.9)
Primary	170 (31.1)	175 (30.8)	54 (34.6)	42 (31.6)	4 (23.5)
Secondary/more	360 (65.9)	375 (66.0)	101 (64.7)	89 (66.9)	12 (70.6)
Own mobile phone, *n* (%)	456 (83.5)	460 (81.0)	135 (86.5)	116 (87.2)	16 (94.1)
Used mobile phone for financial transactions last 12 mo, *n* (%)	379 (69.4)	389 (68.5)	116 (74.4)	96 (72.2)	10 (58.8)
Child’s age, mo^4^^,^^5^	—	—	14.1 (0.4)	14.9 (0.4)	14 (2, 23)
Male child, *n* (%)	—	—	75 (48.1)	60 (45.1)	8 (47.1)
Birth order, *n* (%)					
First born	—	—	57 (36.5)	45 (33.8)	6 (35.3)
Second born	—	—	37 (23.7)	34 (25.6)	4 (23.5)
Third born or later	—	—	62 (39.7)	54 (40.6)	7 (42.2)

1 Missing 20 values for marital status and 2 values for all other characteristics; no statistically significant differences (*P* < 0.05) in characteristics between groups receiving the short and long introduction.

2 Missing 16 values for marital status; no statistically significant differences (*P* < 0.05) in characteristics between groups receiving the short and long introduction.

3 Based on 17 of 19 respondents; characteristics missing for 2 respondents.

4 Mean (standard error) for dietary introduction columns and median (min, max) for cognitive interview column.

5 Child refers to the youngest child in the household.

**FIGURE 2:**
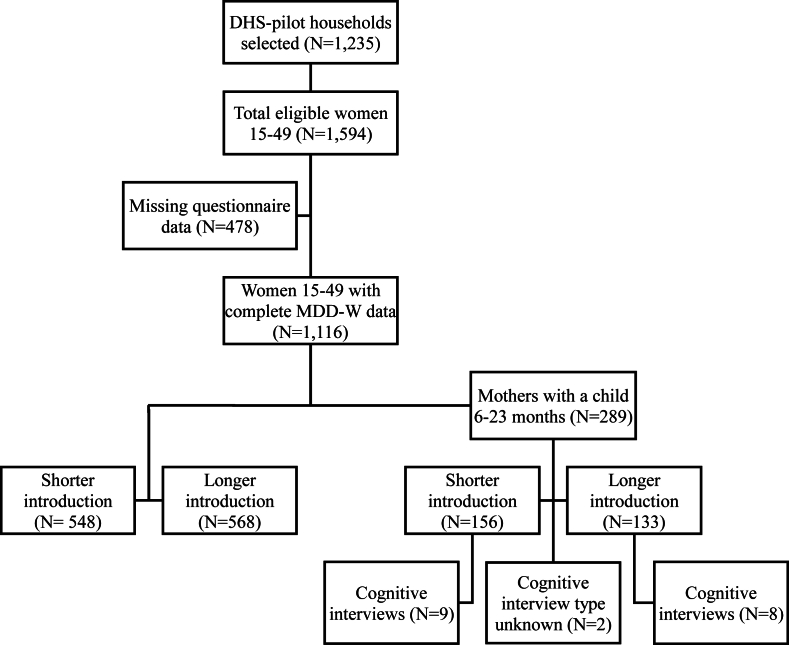
Study flow and sample size. DHS, Demographic and Health Surveys; MDD-W, Minimum Dietary Diversity for Women.

A total of 19 cognitive interviews was conducted among respondents with children under age 2 (Table 1). The cognitive interviews took ∼85 min (median), ranging from 14 to 173 min, to administer. One of the 19 interviews was not completed because the respondent’s child was sick and needed to be taken to the clinic. Figure 2 provides a flow of the study design and associated sample sizes.

### How do instructions influence survey responses to dietary questions?

The proportions for most food groups and the dietary indicators for children and women did not differ significantly between respondents who had received the short introduction and those who had received the long introduction, with 2 exceptions (Table 2). A significantly higher proportion of respondents who had received the short introduction reported consuming dark leafy green vegetables themselves (40.0% compared with 32.6%, *P* ≤ 0.01). Also, a significantly lower proportion who had received the shorter introduction reported that their children had consumed “other vegetables” (22.4% compared with 35.3%, *P* ≤ 0.01).

**TABLE 2:** Comparison of the short and long dietary introduction by dietary indicator and consumption patterns among children and women.

Outcome	Women		Children	
	Short *N* = 548	Long *N*= 568	Short *N* = 156	Long *N* = 133
Minimum dietary diversity	42.5 (38.4, 46.7)	37.0 (33.0, 40.9)	39.7 (32.1, 47.4)	46.6 (38.1, 55.1)
Egg and/or flesh food	—	—	52.6 (44.7, 60.4)	57.1 (48.7, 65.6)
Sweet beverage	75.9 (72.3, 79.5)	78.2 (74.8, 81.6)	78.2 (71.7, 84.7)	72.9 (65.4, 80.5)
Unhealthy food	33.4 (29.4, 37.3)	35.0 (31.1, 39.0)	35.3 (27.8, 42.8)	42.9 (34.4, 51.3)
Foods made from grains	81.9 (78.7, 85.2)	83.6 (80.6, 86.7)	79.5 (73.2, 85.8)	72.9 (65.4, 80.5)
White/pale-fleshed starchy roots and tubers	57.7 (53.5, 61.8)	61.6 (57.6, 65.6)	57.1 (49.3, 64.8)	57.1 (48.7, 65.6)
Beans, peas, lentils	42.2 (38.0, 46.3)	41.2 (37.1, 45.2)	42.3 (34.6, 50.1)	45.9 (37.4, 54.3)
Nuts and seeds	25.9 (22.2, 29.6)	25.9 (22.3, 29.5)	22.4 (15.9, 29.0)	20.3 (13.5, 27.1)
Milk, cheese, yogurt, other milk products	34.3 (30.3. 38.3)	33.6 (29.7, 37.5)	10.3 (5.5, 15.0)	7.5 (3.0, 12.0)
Meat, fish, poultry, organ meats	53.5 (49.3, 57.6)	48.8 (44.7, 52.9)	38.5 (30.8, 46.1)	46.6 (38.1, 55.1)
Eggs	22.8 (19.3, 26.3)	19.2 (16.0, 22.4)	27.6 (20.6, 34.6)	25.6 (18.2, 33.0)
Dark green leafy vegetables	40.0 (35.9, 44.1)^1^	32.6 (28.7, 36.4)^1^	24.4 (17.6, 31.1)	20.3 (13.5, 27.1)
Other vitamin A-rich fruits/ vegetables	29.6 (25.7, 33.4)	30.3 (26.5, 34.1)	23.7 (17.0, 30.4)	26.3 (18.8, 33.8)
Other vegetables	44.0 (39.8, 48.1)	43.5 (39.4, 47.6)	22.4 (15.9, 29.0)^1^	35.3(27.2, 43.5)^1^
Other fruits	31.9 (28.0, 35.8)	32.0 (28.2, 35.9)	31.4 (24.1, 38.7)	25.6 (18.2, 33.0)

1 *P* value <0.01.

Most respondents, whether they had received the short or long introduction, reported no difficulties recalling what they or their children had consumed, and respondents referred to specific memories when describing their process for retrieving information on dietary intake (Table 3). There were no issues identified with the dietary recall period regarding reporting on “yesterday during the day or night” as opposed to “a typical day.” Instructions on the inclusion of “mixed dishes” and exclusion of items consumed in “small quantities” were considered cognitively burdensome, with many respondents not understanding these instructions. Moreover, we found no evidence that respondents’ interpretation of these instructions influenced their responses to the survey.

**TABLE 3:** Key findings and supportive quotes from cognitive interviews on the dietary introduction.

Key findings	Supporting quotes
**Dietary recall period** • Most thought about the previous day and night although a few thought about a different period (e.g. previous 24 h or a typical day). • Most would consider items taken in the middle of the night although giving or taking items at this time was rare. • Most respondents reported no difficulties recalling items they or their child consumed.	• *“I: How did you know that we wanted information about yesterday as opposed to any other day? R: Because you are asking about day and night.”* • *“If she woke up in the middle of the night…Yes [would have included].”* • *“I was thinking about still 24 hours from the time he woke up until now when he woke up.”* • *“Because it is just the previous day I cannot forget.”*
**Items consumed in small quantities** • Most did not recall the instructions, and it was not evident that items would be excluded based on the instructions. • Most demonstrated an understanding of the concept although a few clearly did not. • Some items listed as consumed in small quantities (e.g. carrots) would not be considered small depending on quantity consumed, and there was little agreement on what constitutes a small amount. • It was unclear if respondents reported items used to make sauces or not.	• *“I: When we asked about foods that you and [child name] ate, do you remember any instructions we gave about foods to include and foods to not include. R: Yes. I: What do you remember? R: That I should not include that was added as an ingredient in a small portion.”* • *“The flavors I put in food.”*
**Mixed dishes** • No respondents recalled the instructions, and it was not evident that respondents included foods based on the instructions. • Most had at least a vague understanding of the concept and approximately half demonstrated a clear understanding. • Many were easily able to give examples of mixed and/or composite dishes (e.g. matooke and beans, pumpkin and sweet potato, cassava and beans).	• *“Food combined with other foods, to me, I thought if you are preparing chicken sauce put Irish potato in the meat.”* • *“When we asked you to tell us about the foods you had, you did not mention tomatoes - why not?] I thought you wanted to know if I ate raw tomatoes and onion like salads, I used them [tomatoes] to cook sauce.”* • *“I don’t understand...To say it helps to have a balanced diet, to know if the food I have consumed is balancing is that how I should take it.”*

### Do respondents comprehend terminology used for certain drinks and foods?

In the cognitive interviews, respondents tended to consider “fresh milk” to mean unpasteurized milk, while “packaged” milk connoted pasteurized and branded milk bought from a shop. If asked about milk alone, without further specification, some respondents said they would not think of reporting powdered milk or tinned milk. We found no evidence that respondents incorrectly included condensed milk or nondairy creamers as milk. However, when specifically asked, a few respondents misinterpreted powdered or tinned milk to mean infant formula, and powdered milk to mean composite porridge. When asked what “drinks made with milk” meant, respondents were confused, and some provided examples of solid foods made with milk as an ingredient (Table 4). Some respondents also noted that they had not considered powdered milk, tinned milk, and milk-based drinks when reporting consumption of “drinks made with milk,” which may have been because of low consumption of these drinks among the respondents in peri-urban Kampala, Uganda.

**TABLE 4:** Key findings and supportive quotes from cognitive interviews on terminology.

Original question followed by proposed question in italics^1^	Key findings	Supporting quotes
**Milk and milk-based drinks** Milk from animals, such as fresh, tinned, or powdered milk? *Milk from animals, including fresh, packaged, or powdered milk [such as insert local names for powdered milk, and insert other types of animal milk-based liquids]?*	• Most considered ‘fresh milk’ to be raw cow milk. • Around half indicated they would not consider powdered or tinned milk if asked about milk alone. • Results were mixed on whether powdered milk and tinned milk were different. • There was no mention of condensed milk. • A few considered powdered milk to mean milk mixed with porridge. • A few brand name examples of powdered and tinned milk offered were names of infant formula. • Most did understand the concept of drinks made with milk and several reported incorrect examples, such as baked goods, cheese, ice cream.	*I:“What does the term fresh milk mean to you? R: Fresh milk is milk that has been taken directly from the cow without being taken through the process of the factory and all that. I: What would you call the packaged or the processed milk, are those fresh milk or no? R: To me they are not fresh milk*.” “*Most of the things are made with milk but just added to what is going to be made. It’s added in them, and it becomes one of the items that make the item (cakes, daddy’s, biscuits that’s it).”*
**Yogurt as liquid or solid** Yogurt drinks such as [insert local names of common types of yogurt drinks]? Yogurt other than yogurt drinks? *Did [name] have[Insert general term(s) of specific name(s) of yogurt or yogurt drinks]?* *Did [name] have any [insert general terms or specific names of common types of yogurt or yogurt drinks] as a drink?*	• Most understood (unspecified) yogurt and considered the term to mean both liquid and solid yogurt. • The phrases “yogurt as a drink” and “yogurt, other than yogurt as a drink” were not understood, and either unknowingly responded incorrectly or failed to articulate a definition. • Around half indicated that liquid yogurt could be taken with a spoon and half indicated that solid yogurt could be taken with a straw.	*“I don’t know. ... It’s difficult. ...Because it confuses. Yogurt - there is no yogurt drinks and yogurt.”*
**Sweet or flavored milk and yogurt** If yes: Was the milk a sweet or flavored type of milk? (no change) If yes: Was the yogurt drink a sweet or flavored type of yogurt drink? *If yes: Was it a sweet [or flavored] type of yogurt drink?* (after asking if consumed as drink)	• Around half correctly reported examples or brand names of sweet or flavored milk and yogurt. • Some incorrect examples provided were baked goods, ice cream, powdered milk and/or infant formula (Cow and Gate, Nido), milk flavored at home with cinnamon, and fresh raw milk, goat milk, and cow milk.	*“I: Are they [Jesa brand of milk] sweet and flavored milk? R: Yes, they are. I: You said chocolate. Any other flavors? R: There is chocolate and strawberry.”*
**Sweetened tea, coffee, or herbal drinks** Tea, coffee, or herbal drinks? (no change) If yes: Was the drink sweetened? (no change)	• Nearly all understood the term ‘sweet or sweetened drinks’ as including sugar. A few noted that drinks could be sweetened with honey.	*“If something is sweet, then you must have added something to make it sweet. ... [Such as] sugar. ... Like Honey it can make something sweet.”*
**Fruit juice or fruit-flavored drinks** Fruit juice or fruit-flavored drinks? (no change)	• Around two-thirds differentiated between fruit juice and fruit-flavored drink. • Among those that considered juice and drinks to be different, juice was thought of as fresh, squeezed, and/or made at home (e.g. passion fruit) and drinks were thought of as processed, fake, packaged, and/or soda (e.g. Minute Maid).	*“I think they are different. ... Fruit drink I am going to say they are the processed ones that we usually buy, and the fruit juice is what we make.”*
**Packaged instant noodles** Chips, crisps, puffs, French fries, fried dough, instant noodles, or [insert other commonly consumed ‘sentinel’ fried and salty foods]? (no change)	• Around half correctly described the item, and among the remainder, they generally said they did not know the item.	*“Noodles are those packed things which you just put in water and then boil and then you serve them.”*
**Sweet potato color** Pumpkin, carrots, squash [or sweet potatoes that are yellow or orange inside]? (no change)	• Most understood the color refers to the inside and not the skin. • Some misunderstood ‘fleshed’ to mean cooked potentially because some misunderstood ‘white-fleshed’ for ‘white fresh’ due to the Ugandan accent in English. In Luganda there is no difference in pronunciation between ‘l’ and ‘r’.	*“White sweet potato refers to the] inside because sometimes it can show other color.”*

1 Questions are in the standard form prior to adaptation to country context. Brackets indicate language that is adapted to the country context.

The results also showed that respondents had difficulty distinguishing between liquid and solid yogurt (Table 4). In most cases, respondents considered yogurt to be a solid, semisolid, or soft food; as a result, its consumption was likely appropriately captured in the calculation of minimum meal frequency (data not shown). The concept of sweet or flavored yogurt or milk was only vaguely understood, which may have been due to low consumption of these items among respondents. Conversely, most respondents easily understood the term “sweetened” when used to describe tea or coffee, correctly reported examples of sweetened drinks, and reported correct examples of both fruit juice and fruit-flavored drinks, suggesting that they interpreted these terms as intended. The term “instant noodles” was correctly understood by those who were familiar with the item. Respondents also correctly understood that “sweet potatoes that are yellow or orange inside” referred to the color of the inside of the sweet potato (rather than the outer skin), although the term “flesh” (e.g. “orange-fleshed”) was less commonly understood (Table 4).

### How does question formulation influence accuracy of food classification?

Based on the cognitive interviews, the closed-ended questions did not capture all food items respondents had consumed. Respondents reported the following number of instances of consuming items that were not listed: 21 for dark green leafy vegetables (8 unique items), 12 for other vegetable (4 unique items), 25 for other fruits (12 unique items), 19 for other meat (5 unique items), and 2 for sweet food items (2 unique items) (Table 5). Most of these are included in the country-adapted diet quality questionnaire by the Global Diet Quality Project however, which contains additional food items (often included as two questions for a given food group) [25]. For the open-ended question format, respondents reported items that did not belong in the food group: 4 instances of misclassified items for dark green leafy vegetables (2 unique items), 33 for other vegetables (15 unique items), 6 for other fruits (3 unique items), 5 for other meats (2 unique items), and 30 for sweet foods (20 unique items) (Table 5). The finding that respondents sometimes reported fruits as “other sweet food” may be explained by common understanding of the word “sweet,” which is sometimes understood in Uganda to mean “tasty,” which may have contributed to misreporting of items in this food group.

**TABLE 5:** Foods correctly and incorrectly reported in free listing of foods.

Items	Correct reporting of items not listed in open-ended question^1^	Incorrect reporting of items in open-ended questions
**Dark green leafy vegetables**	*Cowpea leaves (1)*, *jute mallow (1), nightshade leaves (3), pumpkin leaves (2), red amaranth (6), spider plant (5), spinach (1), yam leaves (2).*	Cabbage (3), okra (1).^2^
**Other vegetables**	*African eggplant (5), bitter berries (2), cabbage (4),* French beans (1).	Avocado (1), red amaranth (5), carrot (2), cilantro (1), collard greens (4), cowpea leaves (2), fresh beans (1), green amaranth (4), spider plant leaves (1), nakati [*Solanum aethiopioum*] (5), onion (2), Irish potato (1), pumpkin (1), rice (1), spinach (2).^3^
**Other fruits**	*Apple (6), avocado (2),* berries (1), coconut (1), gooseberries (1), grapes (1), *guava (3), jackfruit (4),* pomegranate (1), *soursop (3),* strawberry (1), *tangerine (1).*	Cucumber (1), lemon (2), sugarcane (2, plus 1 unsure).^4^
**Other meats**	Deer (1), *duck (2), mutton (7), rabbit (4), turkey (5).*	Liver (2), fish (3).^5^
**Sweet foods**	Donuts (1), lollipops (1).	Apple (1), banana (1), bread (2), fried dough (3), jackfruit (2), juice (1), mango (3), maize flour deep fried (2), orange (1), papaya (2), pineapple (2), popcorn (1), Pringles (1), soda (1), steamed dumpling (2), sugar (1), sweet potato (1), watermelon (1), Weetabix (1), yogurt (1).^6^

1 Items listed in *italics* are included in the 2-part question for the food group adapted for Uganda by the Global Diet Quality Project (dietquality.org), so would have been captured using a closed-ended question format. Items not in italics would have been omitted. Two items "ebilyo" and “butula” were unidentified and could not be classified.

2 When directly probed, about half of respondents considered cabbage to be a dark green leafy vegetable (*n* = 8 yes/8 no).

3 Dark green leafy vegetables are asked about immediately prior to other vegetables. Collard greens, cow peas, green amaranth, nakati [*Solanum aethiopioum*], are asked in the dark green leafy vegetable question using open-ended format adapted for Uganda. Carrot and pumpkin are asked about prior to other vegetables, with a question on dark green leafy vegetables between the two. Rice and potato are also asked about earlier in the food lists. When probed directly, about half of respondents considered onion to be an “other vegetable” (*n* = 7 yes/5 no/2 unsure/2 green leafy part of onion). When respondents were asked if they considered onion more like a vegetable or more like a flavoring, more than half of respondents considered it to be a flavoring (*n* = 11 yes/2 no/1 both). Few respondents considered potatoes to be a vegetable (*n* = 2 yes/15 no).

4 Mango (6), passion fruit (4), papaya (5) were also reported, but the question was often posed to respondents in the cognitive interviews as ‘name fruits’ instead of ‘other fruits.’

5 Liver is asked about prior to other meats, with a question on processed meat between the two. Fish is asked about after other meat in the standard questions based on the infant and young child feeding and minimum dietary diversity for women guidelines.

6 The unhealthy food indicator includes a combination of sweet foods and salty and fried foods; thus, salty and fried foods misclassified under sweet foods do not impact the unhealthy food indicator.

Further probing on commonly consumed dark green leafy vegetables or other vegetables (cabbage, onion, and Irish potato) revealed evidence of probable misclassification for some vegetables. When specifically asked, about half of respondents considered cabbage to be a dark green leafy vegetable and onion to be an “other vegetable.” However, when asked to list examples of dark green leafy vegetable and “other vegetables,” few respondents reported either food item. Additionally, the majority of respondents responded that they considered onions to be a flavoring (used in small amounts to flavor food), a view suggesting that it may not be reported as a vegetable (Table 5). Irish potatoes were unlikely to have been misclassified frequently, as few respondents considered Irish potatoes to be a vegetable.

When probing about sweet potatoes, the interviewers found that white and orange sweet potatoes were likely to have been correctly classified, while yellow sweet potatoes were likely to have been misclassified as white. Asking about yellow varieties has the potential to result in overinflation of the vitamin A-rich vegetables; however, overinflation seemed unlikely in this setting, as most respondents did not identify any variety of sweet potato as “yellow.”

### Do respondents comprehend questions on receipt of nutrition services or actions and are they able to recall and retrieve the requested information?

#### Antenatal counseling on maternal diet and IYCF counseling among children under age 2

The antenatal counseling on maternal diet question in which respondents were asked whether a healthcare provider “Talk[ed] with you about which foods or how much food you should eat?” was understood by most respondents, and respondents reported being able to easily recall whether they had received counseling and provided supporting examples that reinforced this. However, respondents were more likely to cite examples of the *types* of foods that should be consumed during pregnancy than the *quantity* of food that should be consumed. Although this question was seeking information about dietary counseling during pregnancy, a few respondents mistakenly responded about dietary counseling received during the postnatal care period. The IYCF counseling question “In the last 6 months, did any healthcare provider or community health worker talk with you about how or what to feed (NAME)?” was also understood by most respondents and it was easy for respondents to recall if they received this service. In free recall, most respondents cited examples of counseling messages received regarding the foods their child should eat, although some reported on the importance of breastfeeding practices, feeding frequency, food consistency, and responsive feeding (Table 6).

**TABLE 6:** Key findings and proposed modifications to survey questions on receipt of nutrition services based on respondent feedback.

Proposed changes to tested question in italics	Key findings	Supporting quotes
Talk with you about which foods *or how much food* you should eat?^1^ (asked during ANC)	• A few respondents’ response to the survey question differed from their response in the cognitive interview. • Most offered examples on what to eat; a few offered examples on how much. • Most reported it was easy to recall.	“*When I went for antenatal care, they were classes and the doctors told us to eat more fruits and vegetables than any other foods.”*
Talk with you about breastfeeding^2^ (ANC) ) (no change)	• A few respondents’ response to the survey question differed from response in cognitive interview. • Most were able to identify the nutrition service correctly, a few responded with regard to postnatal IYCF counseling. • Most reported it was easy to recall.	*“To respond to the question, it wasn’t hard. Because I had received counselling on how to breastfeed when I was pregnant.”*
In the first 2 days after (NAME’s) birth, where did (NAME) stay most of the time during the day and at night, in the same room with you or in a separate room? (asked during PNC) (no change)	• Most were able to identify the nutrition service correctly. • About half could not articulate a definition of ‘most of the time,’ and among those that could, they reported it to mean all the time or a lot of time. • Responses to the survey question did not differ from response in cognitive interview; however, one respondent reported the infant child being taken by a relative for injections the second day after birth (3 h). Among the one respondent who reported not being separated in the survey, they had a cesarean. • Results were mixed on whether responded in reference to the period in the facility, period after discharge from the facility, or both. • Most reported it was easy to recall.	*“Most of the time means were you with him in the same area, in the same space, were you with your baby in the same space? Yes, I was.”*
Observe (NAME) breastfeeding *to see if you are doing it correctly*?^2^ (asked during PNC)	• A few respondents’ response to the survey question differed from their response in the cognitive interview. • Most were able to identify the nutrition service correctly. • Examples provided of someone who might observe breastfeeding included various types of health providers, although one respondent reported elder. When asked directly a few said they would consider a family member. • Most correctly differentiated between passive and active breastfeeding observation. • Most preferred including adding ‘correctly’ to the question. • Most reported it was easy to recall.	*“I choose the one where he/she observed the baby breastfeed correctly. ... Because if a doctor finds the baby is not breastfeeding correctly - he has to check if the baby is in the right position or not and corrects it. But this one who just looks or does not tell you.”*
Tell you where you could get help with breastfeeding?^2^ (asked during PNC) (no change)	• A few respondents’ response to the survey question differed from their response in the cognitive interview. • Most were able to identify the nutrition service correctly. • Majority of sources for help with breastfeeding reported were health facilities and various types of health providers although a few reported peers (e.g. elders, friends, or neighbors). • Most reported it was easy to recall.	*“She told me that if my baby is not breastfeeding like a normal baby, she should rush to the nearest hospital... It was easy because the doctor taught me how to do it and she told me that my baby is feeding well, but I should also continue to see and be keen on how the baby is breastfeeding.”*
In the last 6 mo, did any healthcare provider or community health worker talk with you about how or what to feed (NAME)?^3^ (no change)	• Respondents’ response to the survey question did not differ from response in cognitive interview. • Most were able to identify the nutrition service correctly. • Most were easily able to offer examples regarding what foods to feed and breastfeeding practices while a few also offered correct examples on how to feed.	*“Yeah, they told me I am supposed to give her vegetables, fruits - the bananas, the avocados.”* *“So, since they have a problem of not wanting to eat most of the time, I think it should be how to entice them, the foods you should give them to entice them and how you can have them eat that food in the right time.”*
In the last 3 mo, has any healthcare provider or community health worker measured: a) (NAME)’s weight?, b) (NAME)’s length or height?, c) Around (NAME)’s upper arm? SHOW IMAGE OF MUAC TAPE^3^^,^^4^ (no change)	• Most were able to identify the nutrition service correctly.	*“The weight, yes... they tell to measure with the baby then subtract yours. Measure with the baby, [then] measure alone then subtract.”*

Abbreviations: ANC, antenatal care; MUAC, mid-upper arm circumference; PNC, postnatal care; IYCF, infant and young child feeding.

1 Part of a multipart question: “As part of your antenatal care during this pregnancy, did a healthcare provider do any of the following:”.

2 Part of a multipart question: “During the first 2 days after (NAME)’s birth, did any health care provider do the following:”.

3 Not possible to measure ease of recall due to wording in the cognitive interview guide.

4 Not possible to measure discordance between survey question and cognitive interview response due to wording in the cognitive interview guide.

#### Breastfeeding counseling during antenatal and postnatal care and breastfeeding referral and observation of breastfeeding during postnatal care

Respondents were able to easily recall, and most understood the questions on, breastfeeding during the antenatal and postnatal care period (“Talk with you about breastfeeding?”). For the question on breastfeeding referral (“Tell you where you could get help with breastfeeding?”), although many respondents did not receive breastfeeding referral as part of postnatal care, they were able to name places where new mothers can go to find help with breastfeeding, indicating that the question was well understood. Nearly all respondents expressed confidence that they would have been able to recall a referral if they had received one (Table 6).

Respondents also reported being able to easily recall whether a healthcare worker had observed them breastfeeding in the period after delivery. When asked for an example of someone who might have observed them breastfeeding, most respondents identified appropriate providers of skilled breastfeeding support. Also, respondents were able to differentiate between passive and active breastfeeding observation. Inclusion of the term “correctly” in the question “Observe (NAME) [correctly] breastfeeding?” was preferred by respondents because it made the intent of the question clearer, i.e. the health worker was observing breastfeeding for the purpose of guidance. After the cognitive interviews were completed, the issue was raised that respondents might interpret “correctly” to mean that the mother was correctly breastfeeding. Thus, additional language “to see if you are doing it correctly,” while not directly tested, may be a better formulation of the question (Table 6).

#### Rooming-in postdelivery

For the question, “In the first two days after (NAME’s) birth, where did (NAME) stay most of the time during the day and at night, in the same room with you or in a separate room?”, respondents reported being able to easily recall where they and their newborn were during the first 2 d after delivery. Respondents had difficulty *articulating* the meaning of “most of the time,” although they did not report difficulty in understanding it. When elaborating on the experiences they referenced while responding to the survey question about periods of separation, nearly all respondents correctly identified the relevant information and responded accordingly (Table 6).

#### Growth monitoring

For the question, “In the last 3 months, has any healthcare provider or community health worker measured:” with the response options of weight, length or height, or upper arm, respondents understood this question was referring to growth monitoring. Respondents were shown a picture of a mid-upper arm circumference (i.e. MUAC) tape to assist them in answering the option “upper arm.” Respondents were also able to identify that the question was asking about measurements in a community or facility setting, even though in this study context, this service seemed to be available only in the facilities (Table 6).

### Does social desirability bias influence survey responses?

The potential for social desirability bias was explored for scenarios of exclusive breastfeeding in the first 2 d (i.e. prelacteal feeding), feeding of soda to infants, and feeding of meat to infants. Although many respondents noted that they themselves would answer these questions honestly, many also indicated that other people may be dishonest in answering these questions. When asked if people would answer honestly to the question on giving their child anything other than breastmilk to eat or drink in the first 2 d after birth, approximately half of respondents thought other people would not respond honestly. As explained by one respondent, *“No... Because some give…because of the rituals and they don’t want [that] to be said.”* Similarly, about half of respondents reported that others would underreport the consumption of soda, as noted by a respondent: *“Will not respond [honestly] because medical personnel told us not to take soda.”* A few respondents indicated that they or others might also knowingly misreport meat consumption. Misreporting was hypothesized by respondents to occur in both directions: overreporting, because meat is considered a status food, and underreporting because some considered meat to be unhealthy.

## Discussion

As part of The DHS Program’s pilot study in Uganda, we used quantitative data and results of cognitive testing to assess whether respondents’ interpretation of and responses to survey questions on dietary intake and receipt of nutrition services were meeting the questions’ intended constructs consistently and correctly. We identified sources of response error, as well as modifications that may improve clarity and elicit responses in the intended manner, in 3 areas: revisions to terminology and wording of dietary intake and receipt of nutrition services questions, sequencing of food lists, and considerations for use of open- compared with closed-ended food lists.

Regarding dietary intake, we found no evidence that a long introduction to the questions added any benefit over a short introduction, as also shown elsewhere [29]. Based on analysis of cognitive interviews, the long introduction did not appear to improve retrieval cues that activated respondents’ memories or facilitated respondents to consider the previous day rather than a typical day. To explain this result, it is possible that respondents paid less attention to the long introduction or that interviewers omitted portions of it, but this possibility was not examined in this study. However, other research has suggested that using question stems to periodically remind respondents of the recall period (e.g. “Yesterday, did you have any of the following foods?”) may help improve the comprehension of instructions or the quality of responses [30]; this strategy has been used in the Gallup World Poll, a multitopic survey [25].

Regardless of which introduction respondents received, we found that instructions on the inclusion of items in mixed dishes and exclusion of items consumed in small quantities were cognitively burdensome, with evidence of case-by-case judgment in the interpretation of the instructions. These results suggest that these instructions are unlikely to improve reporting, as also found by others in different country contexts [29]. Well-adapted food lists that include items consumed in sufficient quantities from mixed dishes and sauces and exclude items consumed in small quantities may at least partially alleviate the challenge of respondents not being able to understand the instructions [7]. Alternative approaches for improving reporting include using photographs of mixed dishes and portion sizes [31]. The benefit of additional visual aids would need to be weighed against the importance of keeping data collection simple.

Regarding terminology of drink and food items, most respondents recognized and interpreted terms on drinks and foods in questions as intended or required only small clarifications in wording. The exceptions were milk, which required revised terminology, and yogurt, which required restructuring because respondents did not distinguish between liquid and solid yogurt, which was also found in other settings by Herforth et al. [9]. The yogurt questions were therefore restructured to first ask respondents whether they had taken any yogurt and then ask about whether it was a drink and whether it was sweetened or flavored. Some respondents were unable to distinguish between all-natural juice and fruit-flavored drinks, which provides evidence in support of the sweet beverage indicator being constructed to include both types of drinks [4].

We saw evidence that misclassification occurred when respondents were asked to categorize foods in open-ended questions. In some cases, misclassification could have directional effects on the survey results. For example, misreporting cabbage as a dark green leafy vegetable or sugar cane as a fruit may inflate the minimum dietary diversity indicator, or misclassification of fruits as sweet foods may inflate the unhealthy feeding indicator. In some cases, misclassification may be fully or partially mitigated by placing commonly misclassified foods in preceding questions [32]. In our study, some respondents considered fish to be a type of meat; thus, when an open-ended format is used to ask about meat, the question on fish could be placed before the question on meat to avoid double counting. Open-ended questions could also have resulted in underreporting, if respondents could not think of other items that were not listed, which was observed by Herforth et al. [30]. However, this study did not examine that possibility.

Use of comprehensive country-specific food list adaptations that are publicly available in 140 countries increases the utility of using closed-ended questions that list specific foods to avoid misclassification; DHS, Gallup World Poll, Living Standards Measurement Study (LSMS), and USAID Feed the Future program all use these question adaptations for improved comparability [9,25]. While the closed-ended format used in our study did not include all food items consumed by respondents, most notably for other fruits and dark green leafy vegetables, the omission of less commonly consumed food items might not result in underreporting of the food group if other more common items in the same food group are reported. A study in China showed the list-based approach using closed-ended questions for dark green leafy vegetables, other vegetables, and other fruits compared with the multiple pass 24-h quantitative recall method captured 95% or more of people who consumed each food group at the national level and in most provinces [33]. Furthermore, at least in our study setting, including 2 closed-ended questions for food group with a wide diversity of foods consumed would capture nearly all items [25]. Closed-ended questions do not perfectly capture all consumption, but given the amount of misreporting we observed in open-ended questions, the closed-ended approach appears preferable. Although a potential increase in respondent fatigue (with its associated risks) by including more food list questions is an important consideration, especially in multitopic surveys, and it requires further exploration.

Nutrition counseling and breastfeeding referral, rooming-in postdelivery, and growth monitoring questions have not routinely been collected in population-based surveys. This study demonstrated that, overall, respondents understood these services as originally worded and were able to recall the required information, with a few exceptions. To determine whether rooming-in occurred postdelivery, respondents were asked if the child stayed with the mother “most of the time,” and some respondents had difficultly articulating this concept. Nevertheless, in a facility setting, there are usually 2 extremes: “rooming-in” where mom and infant are kept together routinely, or nursery care where they stay separate [34]. Thus, the practice of rooming-in may be able to be correctly captured using the survey question tested because it does not require a high degree of sensitivity. A second exception was proposed modifications to the question on observation of breastfeeding to reflect that a healthcare provider should be not just observing but also assessing whether the correct approach is being used to breastfeed. Our revised wording for this question is similar, but with further adjustments, to that proposed previously by others [16].

Questions about receipt of nutrition services tested whether counseling was received at key time points; respondents were not asked about the content and quality of counseling because population-based surveys are not suitable for collecting this information [15]. However, for the question on receipt of IYCF counseling, respondents were asked about “what” and “how” to feed their child. During cognitive testing, we found respondents were more likely to report on “what,” as found elsewhere [16]. The reason for this is unknown (e.g. it could be easier to remember or to understand the “what”), as is whether this impacts the validity of the receipt of counseling indicator.

Although results of studies on the validity of questions on receipt of nutrition services in population-based surveys have been mixed, these types of surveys provide representative, and often the only, data on receipt of nutrition services in many LMIC settings. Alternative data sources, such as facility-based surveys and administrative data, allow for more direct observation and shorter recall periods but often do not capture community-based services. Also, data collected through administrative systems can overburden health workers. All data sources inherently have limitations and measurement error, and this should be accounted for in the interpretation of findings and in the cross-linking of data systems.

We found some indication of social desirability bias in the downward direction for giving liquids other than breastmilk in the first 2 d and for soda consumption, as seen elsewhere [18,19,21]. In alignment with others, practices that were perceived to be less or more socially desirable appeared to be influenced by exposure to dietary messages [35]. We relied on asking respondents if they thought individuals would answer questions honestly rather than using more rigorous methods, such as a social desirability scale [36] or comparing self-reported dietary intake against a reference method [19]. Nevertheless, our findings indicate the importance of training interviewers to establish good rapport, remain neutral, and conduct interviews privately to attenuate social desirability bias [37].

### Strengths and limitations

Because The DHS Program’s pilot study was designed to mimic a real-world survey context, our cognitive testing results are directly applicable to DHS surveys, one of the main sources of nutrition data globally. Based on the findings of this study, The DHS Program has revised its standard questionnaire, highlighting the value of cognitive testing as a more routine part of developing tools for large-scale surveys. The results can also help inform question formulation and interpretation of results for other survey types. Additional cognitive testing of survey instruments prior to implementation that still account for the need for comparability between surveys may also be beneficial. When survey length is less of a concern, there may be the opportunity to provide more questions.

This study was conducted in peri-urban Greater Metropolitan Kampala, where many respondents had at least some secondary or higher education. The results may not be generalizable to other locations, even other peri-urban locations in Uganda. For example, classification of potatoes as a staple food in Uganda differs from its popular classification as a vegetable in South Asia [29], and the understanding of terms such as “tinned milk” or “instant noodles” in this population may differ from terminology for the same item in other settings [9]. While this study shed light on how respondents understood and processed the survey questions studied, it also pointed to the need for similar research and careful question adaptation in other countries, such as described in Herforth et al. [9,30]. Studies such as this need to be seen as a part of a continuing process of improvement rather than a conclusive answer for all settings.

Although the cognitive interview method provides deep insights difficult to achieve via other methods, the cognitive interview method requires highly skilled researchers with topical expertise (e.g. nutrition) who are also fluent in local languages [38]. The verbal probing technique used elicits specific information but requires greater interviewing skills than the think-aloud method. The cognitive interviews for this study were carried out by a team including a highly skilled researcher with topical expertise and a local interviewer with postgraduate study in psychology, previous research experience, and fluency in the local language (Luganda). In this study, cognitive training comprised about 3 d of classroom lecture, practice, and role play and 1 d of field practice. The investment requirements for cognitive interviewing likely makes it infeasible for routine implementation. Where possible, however, the deep insights gained underscore the importance of cognitive interviews during the development of questions for new indicators and constructs, especially for large-scale population-based studies, so that we can answer with greater certainty, “Did we get it right?”

## Conclusion

Global and country entities rely on accurate survey data to monitor and evaluate policies and programs. Our study fills important research gaps on the operationalization of new and recently revised guides on the collection of IYCF, minimum dietary diversity for women, and receipt of nutrition services indicators. Our findings indicate a need to update guidance on some standard questions. There remains a need for greater investment in research on the “how to” of formulating and administering nutrition survey questions.

## Author contributions

The authors’ responsibilities were as follows—AH, SN: designed the research study; AH, SN, ALSB: developed the research instruments; ALSB: trained the data collectors and conducted the cognitive interview research; ALSB: conducted the qualitative analysis; AH, SN: reviewed the analysis; SN: conducted the quantitative analysis;. SN: wrote the paper with substantial input from AH and ALSB; SN: had primary responsibility for final content; SN: project administration and funding acquisition; and all authors: read and approved the final manuscript.

## Data availability

Data described in the manuscript, code book, and analytic code will be made available upon request.

## Funding

The study was implemented with support from the United States Agency for International Development (USAID) and the Bill & Melinda Gates Foundation through The DHS Program (#720-OAA-18C-00083). The views expressed are those of the authors and do not necessarily reflect the views of USAID or the United States Government.

## Conflict of interest

The authors report no conflicts of interest.

## References

[1] Victora C.G., Christian P., Vidaletti L.P., Gatica-Domínguez G., Menon P., Black R.E. (2021). Revisiting maternal and child undernutrition in low-income and middle-income countries: variable progress towards an unfinished agenda. Lancet.

[2] Gillespie S., Menon P., Heidkamp R., Piwoz E., Rawat R., Munos M. (2019). Measuring the coverage of nutrition interventions along the continuum of care: time to act at scale. BMJ Glob. Health.

[3] FAO (2021).

[4] WHO and UNICEF (2021).

[5] DataDENT (2021).

[6] The Demographic and Health Survey Program. [cited 21 July, 2022]. Available from: https://dhsprogram.com/.

[7] Martin-Prevel Y., Becquey E., Arimond M. (2010). Food group diversity indicators derived from qualitative list-based questionnaire misreported some foods compared to same indicators derived from quantitative 24-hour recall in urban Burkina Faso. J. Nutr..

[8] Gibson R.S., Charrondiere U.R., Bell W. (2017). Measurement errors in dietary assessment using self-reported 24-hour recalls in low-income countries and strategies for their prevention. Adv. Nutr..

[9] Herforth A.W., Sokourenko K., Gonzalez B.C., Uyar B.T.M., Bulungu A.L.S., Vogliano C. (2024). Adaptation of the Diet Quality Questionnaire as a global public good for use in 140 countries. Curr. Dev. Nutr..

[10] Uyar B.T.M., Talsma E.F., Herforth A.W., Trijsburg L.E., Vogliano C., Pastori G. (2023). The DQQ is a valid tool to collect population-level food group consumption data: a study among women in Ethiopia, Vietnam, and Solomon Islands. J. Nutr..

[11] Bryce E., Katz J., Heidkamp R., Lama T.P., Khatry S.K., LeClerq S. (2022). Validation of maternal report of nutrition-related interventions and counselling during antenatal care in southern Nepal, Matern. Child Nutr..

[12] McKay M., Munos M.K., Kim S.S., Bryce E., Bucina H., Marchant T. (2024). Assessing the validity of maternal report on breastfeeding counselling in Kosovo’s primary health facilities. BMC Pregnancy Childbirth.

[13] McCarthy K.J., Blanc A.K., Warren C., Bajracharya A., Bellows B. (2020). Validating women’s reports of antenatal and postnatal care received in Bangladesh, Cambodia and Kenya. BMJ Global Health.

[14] McCarthy K.J., Blanc A.K., Warren C.E., Mdawida B. (2018). Women’s recall of maternal and newborn interventions received in the postnatal period: a validity study in Kenya and Swaziland. J. Glob. Health.

[15] Kim S.S., Ashok S., Avula R., Mahapatra T., Gokhale P., Walton S. (2023). Moderate accuracy of survey responses about infant and young child feeding counseling reported by mothers with children less than 1 year of age in India. J Nutr.

[16] Ashok S., Kim S.S., Heidkamp R.A., Munos M.K., Menon P., Avula R. (2022). Using cognitive interviewing to bridge the intent-interpretation gap for nutrition coverage survey questions in India, Matern. Child Nutr..

[17] Thorne-Lyman A.L., Lama T.P., Heidkamp R.A., Manandhar P., Subedi S., Munos M.K. (2022). Cognitive testing of questions about antenatal care and nutrition interventions in southern Nepal. Soc. Sci. Med..

[18] Börnhorst C., Huybrechts I., Ahrens W., Eiben G., Michels N., Pala V. (2013). Prevalence and determinants of misreporting among European children in proxy-reported 24 h dietary recalls. Br. J. Nutr..

[19] Hebert J.R., Hurley T.G., Peterson K.E., Resnicow K., Thompson F.E., Yaroch A.L. (2008). Social desirability trait influences on self-reported dietary measures among diverse participants in a multicenter multiple risk factor trial. J. Nutr..

[20] Hebert J.R., Ma Y., Clemow L., Ockene I.S., Saperia G., Stanek E.J. (1997). Gender differences in social desirability and social approval bias in dietary self-report. Am. J. Epidemiol..

[21] Mulol H., Coutsoudis A. (2018). Limitations of maternal recall for measuring exclusive breastfeeding rates in South African mothers. Int. Breastfeed. J..

[22] Li R., Scanlon K.S., Serdula M.K. (2005). The validity and reliability of maternal recall of breastfeeding practice. Nutr. Rev..

[23] Tourangeau R., Jabine T.B., Straf M.L., Tanur J.M., Tourangeau R. (1984). Cognitive Aspects of Survey Methodology: Building a Bridge between Disciplines.

[24] Tourangeau R., Rips L.J., Rasinski K. (2000).

[25] Global Diet Quality Project. Country-Adapted Diet Quality Questionnaires. [cited 18 November, 2024]. Available from: https://www.dietquality.org/.

[26] Shiyanbola O.O., Bolt D., Tarfa A., Brown C., Ward E. (2019). A content validity and cognitive interview process to evaluate an Illness Perception Questionnaire for African Americans with type 2 diabetes. BMC Res. Notes..

[27] Hannan A., Heckert J., James-Hawkins L., Yount K.M. (2020). Cognitive interviewing to improve women's empowerment questions in surveys: application to the health and nutrition and intrahousehold relationships modules for the project-level Women’s Empowerment in Agriculture Index, Matern. Child Nutr..

[28] Zarnowiecki D., Byrne R.A., Bodner G.E., Bell L.K., Golley R.K. (2020). Improving the reporting of young children’s food intake: insights from a cognitive interviewing study with mothers of 3-7-year old children. Nutrients.

[29] Khadka S., Sharma N., Yuen-Esco K., Pries A.M. (2024). Cognitive interviewing to improve infant and young child dietary assessments in the Nepal Demographic and Health Survey. Curr. Dev. Nutr..

[30] Herforth A.W., Sattamini I.F., Olarte D.A., Diego-Rosell P., Rzepa A. (2024). You say potato, I say vegetable; you say tomato, I say fruit: cognitive validity of food group-based dietary recall questions. Curr. Dev. Nutr..

[31] Bulungu A.L.S., Palla L., Priebe J., Forsythe L., Katic P., Varley G. (2021). Validation of a life-logging wearable camera method and the 24-h diet recall method for assessing maternal and child dietary diversity. Br. J. Nutr..

[32] Neelakantan N., Whitton C., Seah S., Koh H., Rebello S.A., Lim J.Y. (2016). Development of a semi-quantitative food frequency questionnaire to assess the dietary intake of a multi-ethnic urban Asian population. Nutrients.

[33] Ma S., Herforth A.W., Vogliano C., Zou Z. (2022). Most commonly-consumed food items by food group, and by province, in China: implications for diet quality monitoring. Nutrients.

[34] Pérez-Escamilla R., Martinez J.L., Segura-Pérez S. (2016). Impact of the Baby-friendly Hospital Initiative on breastfeeding and child health outcomes: a systematic review, Matern. Child Nutr..

[35] Miller T.M., Abdel-Maksoud M.F., Crane L.A., Marcus A.C., Byers T.E. (2008). Effects of social approval bias on self-reported fruit and vegetable consumption: a randomized controlled trial. Nutr. J..

[36] Strahan R.F. (2007). Regarding some short forms of the Marlowe-Crowne Social Desirability Scale. Psychol. Rep..

[37] Krumpal I. (2013). Determinants of social desirability bias in sensitive surveys: a literature review. Qual. Quant..

[38] Beatty P.C., Willis G.B. (2007). Research synthesis: the practice of cognitive interviewing. Public Opin. Q..

