# Supplementary material for: Cognitive Testing of Dietary Assessment and Receipt of Nutrition Services for Use in Population-Based Surveys: Results from a Demographic and Health Surveys Pilot in Uganda
Source: Curr Dev Nutr. 2024 Nov 30;9(Suppl 1):104518. doi: 10.1016/j.cdnut.2024.104518 (PMC12125690; doi:10.1016/j.cdnut.2024.104518)
Supplement: Multimedia component 1 [file mmc1.docx]

**Supplementary file**

**Supplementary Table 1: Nutrition Cognitive Interview Guide**

| ◼ | Indicates that the question should be asked to all respondents | | | | | ★ | Indicates that the question is conditional. | |
| --- | --- | --- | --- | --- | --- | --- | --- | --- |
| **Baby's name:** | | | | | **Start time:** | | | |
| **CI#** | | **DHS#** | **Type** | **English** | | | |  |
|  | |  | ◼ | - Thank you for your responses so far! - Now we're going to shift to the second part of the interview. I'm going to ask you about your understanding of the questions I have just asked. - For these questions, there are no right or wrong answers. We want your feedback to make sure that these questions are easy for other mothers like you to understand. - This part of the interview will be more of a conversation. There may be some questions I ask that don't make sense - let me know and I will rephrase. - If it's OK, this part of the interview will be recorded. [WAIT FOR CONSENT] - Do you have any questions before we start? [ADDRESS ANY QUESTIONS] - I am going to turn the recorder on now. [TURN ON RECORDER] | | | |  |
| 1.a | |  | ◼ | - When you were telling me about the foods or drinks that you consumed, what period of time you were thinking about? - When you were telling me about the foods or drinks that (NAME) consumed, what period of time you were thinking about?   OTHER PROBES, IF NEEDED:   - To clarify, when you or (NAME) said that you ate [SELECT A FEW EXAMPLES OF FOODS SHE REPORTED EATING], were you thinking specifically about yesterday or were you thinking about a typical day? | | | |  |
| 1.b | |  | ★ | IF YESTERDAY: Thank you for telling me about the foods/drinks that you and (NAME) had yesterday.   - How did you know that I wanted information about yesterday as opposed to any other day?   [PROBE IF RESPONSE FOR CHILD AND WOMAN DIFFERED IN 1A] | | | |  |
| 1.c | |  | ★ | IF A TYPICAL DAY:   - What does a typical day mean to you?   OTHER PROBES, IF NEEDED:   - What does "yesterday during the day or at night" mean to you? - Why did you respond with foods for a typical day instead of yesterday? - How are the foods you ate yesterday different from the foods you eat on a typical day? - How are the foods (NAME) ate yesterday different from the foods (NAME) eats on a typical day?   [PROBE IF RESPONSE FOR CHILD AND WOMAN DIFFERED IN 1A] | | | |  |
| 1.d | | INTRO: LONG  SHORT  (CIRCLE ONE) | ★ | IF LONG:   - When I asked you to think about the first thing you and (NAME) ate or drank in the morning and where you were when you had a food or drink in the middle of the day, the evening, and the night: Did that help you think about yesterday as opposed to any other day? Why? | | | |  |
| 1.e | |  | ◼ | How sure were you about the foods and drinks you had yesterday?  OTHER PROBES, IF NEEDED:   - Do you remember the foods and drinks you had yesterday very well, or not so well? | | | |  |
| 1.f | |  | ★ | IF UNSURE:   - What were you unsure about? | | | |  |
| 1.g | |  | ◼ | How sure were you about the foods and drinks (NAME) had yesterday?  OTHER PROBES, IF NEEDED:   - Do you remember the foods and drinks (NAME) had yesterday very well, or not so well? | | | |  |
| 1.h | |  | ★ | IF UNSURE:   - What were you unsure about? | | | |  |
| 2.a | |  | ◼ | Does (NAME) take food or drinks other than breastmilk during the night?  OTHER PROBES, IF NEEDED:   - Does (NAME) ever wake up in the middle of the night and take food or drinks other than breastmilk? | | | |  |
| 2.b | |  | ★ | IF YES:   - What foods or drinks other than breastmilk does (NAME) take during the night? - When I asked whether (NAME) had any food or drink "during the day or at night”, were you thinking that meant until (NAME) went to sleep or were you also thinking about foods or drinks (NAME) had during the night? | | | |  |
| 2.c | |  | ★ | IF NO:   - If (NAME) had taken food or drink during the night, would you have reported it? | | | |  |
| 3.a | |  | ◼ | When I asked about the foods you and (NAME) ate, do you remember any instructions I gave about foods to include and foods to NOT include? | | | |  |
| 3.b | |  | ★ | IF YES: [WITHOUT REMINDING HER OF THE WORDING]   - What do you remember about the foods I instructed you to include? - What do you remember about the foods I instructed you to NOT include? | | | |  |
| 4.a | |  | ◼ | I said, "do not answer ‘yes’ for any food or ingredient only used in a small amount to add flavor to a dish".   - What do you think I meant by a "food or ingredient only used in a small amount to add flavor"?   OTHER PROBES, IF NEEDED:   - Can you name some foods or ingredients that you only use in a small amount to add flavor to a dish? - To verify, you said that you use [FOOD] in a small amount to add flavor to a dish? - When you use [FOOD] in a small amount to add flavor, how much [FOOD] do you use? - Did you eat any [FOOD] yesterday? [VERIFY IF THESE WERE REPORTED - PROBE ACCORDINGLY] - Did (NAME) eat any [FOOD] yesterday? [VERIFY IF THESE WERE REPORTED - PROBE ACCORDINGLY] - Are there any other foods or ingredients you used yesterday in small amount but did not report? | | | |  |
| 4.b | |  | ◼ | - Were there any foods that you ate that you did not report because they were only "used in a small amount to add flavor"? - Were there any foods that (NAME) ate that you did not report because they were only "used in a small amount to add flavor"? | | | |  |
| 4.c | |  | ★ | IF YES WOMAN:   - What was the food? - What dish was it in? - Why did you consider it to be “a small amount to add flavor”?   IF YES CHILD:   - What was the food? - What dish was it in? - Why did you consider it to be “a small amount to add flavor”? | | | |  |
| 5.a | |  | ◼ | I asked that you include food “even if it was combined with other foods”.   - What do you think I meant by food "combined with other foods"?   OTHER PROBES, IF NEEDED:   - Can you name some foods that are combined with other foods? | | | |  |
| 5.b | |  | ◼ | - Name some foods "combined with other foods" that you ate yesterday. - Name some foods "combined with other foods" that (NAME) ate yesterday   [VERIFY IF THESE WERE REPORTED - PROBE IF NOT] | | | |  |
| 6.a | |  | ◼ | If I asked if (NAME) had milk: Would you think of anything other than cow milk? | | | |  |
| 6.b | |  | ◼ | If I asked if (NAME) had milk: Would you have considered “tinned milk”?   - Does (NAME) ever drink “tinned milk” - What does “tinned milk” mean to you? [PROBE CONDENSED MILK]   OTHER PROBES, IF NEEDED:   - Where do you get “tinned milk”? - What kind of packaging does “tinned milk” come in? - Is “tinned milk” wet or dry? - Are there any brand names you think about when you think about "tinned milk"? | | | |  |
| 6.c | |  | ◼ | If I asked if (NAME) had milk: Would you have considered “powdered milk”?   - Does (NAME) ever drink “powdered milk” - What does “powdered milk” mean to you? [PROBE CONDENSED MILK]   OTHER PROBES, IF NEEDED:   - Where do you get “powdered milk”? - What kind of packaging does “powdered milk” come in? - Is “powdered milk” wet or dry? - Are there any brand names you think about when you think about "powdered milk"? | | | |  |
| 6.d | |  | ◼ | Are “tinned milk” and “powdered milk” the same thing or different things? | | | |  |
| 6.e | |  | ★ | IF DIFFERENT:   - What is the difference between “tinned milk” and “powdered milk”? | | | |  |
| 6.f | |  | ◼ | - Does (NAME) ever drink “fresh milk” - What does “fresh milk” mean to you?   OTHER PROBES, IF NEEDED:   - Where do you get “fresh milk”? - What kind of packaging does “fresh milk” come in? - How do you considered milks that are liquid but come pre-packaged - are they "fresh milk" or no? | | | |  |
| 7.a | |  | ◼ | What drinks made with milk are available in your community? PROBE: Any others? [PROBE MILKSHAKE, IF NOT STATED] | | | |  |
| 7.b | |  | ◼ | Are there sweet or flavored milks available in your community?   - IF YES: What type of sweet or flavored milks are available? | | | |  |
| 7.c | |  | ◼ | When I asked if (NAME) had “Milk from animals, such as fresh, tinned or powdered milk, including milk tea?”, did you consider any other types of drinks that were made from milk or not? | | | |  |
| 8.a | |  | ◼ | - Does (NAME) ever drink “fruit juice”? - Do you ever drink “fruit juice”? - What “fruit juices” are available in your community? PROBE: Any others? | | | |  |
| 8.b | |  | ◼ | - Does (NAME) ever drink “fruit drinks”? - Do you ever drink “fruit drinks”? - What “fruit drinks” are available in your community? PROBE: Any others? | | | |  |
| 8.c | |  | ◼ | Are “fruit juice” and “fruit drinks” the same thing or different things? | | | |  |
| 8.d | |  | ★ | IF DIFFERENT:  What is the difference between “fruit juice” and “fruit drinks”? | | | |  |
| 9. | |  | ◼ | I asked if you or (NAME) had soda. Do you think most people would answer this question honestly? Why? | | | |  |
| 10.a | |  | ◼ | I asked if (NAME) had “tea or coffee”. I then asked if it was “sweetened”. What do you think I meant by “sweetened”? | | | |  |
| 10.b | | 636h  SWEETENED | ★ | IF YES TO 636h:  What did you use to sweeten it? | | | |  |
| 11.a | | SWEETENED  636j | ★ | IF YES TO 636j SWEETENED:  I asked whether your child had any other liquids that were sweetened, and you said yes.   - What sweetened drink(s) did (NAME) have yesterday? - What did you use to sweeten it? | | | |  |
| 11.b | | SWEETENED  643w | ★ | IF YES TO 643w SWEETENED:  I asked you whether you had any other drinks that were sweetened, and you said yes.   - What sweetened drink(s) did you have yesterday? - What did you use to sweeten it? | | | |  |
| 12.a | |  | ◼ | Now I'm going to ask you a new question: Yesterday during the day or night did (NAME) have yogurt or bongo?   - Is this question easy or difficult to answer? Why? | | | |  |
| 12.b | |  | ★ | IF YES:   - Did (NAME) take the yogurt or bongo as a drink? - Is this question easy or difficult to answer? Why? | | | |  |
| 12.c | |  | ★ | IF YES:   - Was the yogurt or bongo drink sweet or flavored? - Is this question easy or difficult to answer? Why? | | | |  |
| 13.a | |  | ◼ | Earlier I asked, "Yesterday during the day or at night, did (NAME) drink ... yogurt drink or bongo as a drink".   - What do you think I meant by "yogurt drink or bongo as a drink”? - Is this question easy or difficult to answer? Why?   OTHER PROBES, IF NEEDED:   - What does it mean to take yogurt "as a drink"? | | | |  |
| 13.b | |  | ◼ | What sweet or flavored type of yogurt drink or bongo as a drink are available in your community? PROBE: Any others?   - How are these drinks taken? [PROBE: STRAW, CUP, SPOON, ETC. TO VERIFY "DRINKS"] | | | |  |
| 13.c | |  | ◼ | I also asked, "Yesterday during the day or at night, did (NAME) eat ... yogurt, other than yogurt drinks".   - What do you think I meant by "yogurt, other than yogurt drinks"? - Is this question easy or difficult to answer? Why? | | | |  |
| 14.a | | 637d:  643c: | ◼ | I asked if you had “Irish, boiled cassava, yam, matooke, plantain, white sweet potato, cassava bread, or kivuvu”.   - Name some foods based on these instructions. | | | |  |
| 14.b | |  | ◼ | What does “white sweet potato” mean to you? | | | |  |
| 14.c | |  | ◼ | When thinking about a “white sweet potato”, do you think about: (1) the flesh on the inside of the sweet potato, or (2) the skin on the outside of the sweet potato, or (3) something else? | | | |  |
| 14.d | |  | ◼ | What does “white fleshed sweet potato” mean to you? | | | |  |
| 14.e | |  | ◼ | Are “white sweet potatoes” and “white fleshed sweet potatoes” the same thing or different things? | | | |  |
| 14.f | |  | ★ | IF DIFFERENT:  What is the difference between “white sweet potato” and “white fleshed sweet potato”? | | | |  |
| 14.g | |  | ◼ | Which term - “white sweet potato” and “white fleshed sweet potato” - makes more sense to you? Why? | | | |  |
| 15.a | |  | ◼ | [PHOTO1: “LIGHT YELLOW” NON-BIOFORTIFIED S.P.]   - What color sweet potato would you consider this? | | | |  |
| 15.b | |  | ◼ | [PHOTO2: “DARKER YELLOW/ORANGE” BIOFORTIFIED S.P.]   - What color sweet potato would you consider this? - Have you ever seen a sweet potato like this before? | | | |  |
| 15.c | |  | ◼ | [PHOTO3: “LIGHT YELLOW” BIOFORTIFIED S.P.]   - What color sweet potato would you consider this? | | | |  |
| 15.d | |  | ◼ | [PHOTO4: “WHITE” NON-BIOFORTIFIED S.P.]   - What color sweet potato would you consider this? | | | |  |
| 15.e | |  | ★ | IF REPORTED THE YELLOW S.P. AS WHITE:   - Do you have yellow sweet potatoes in your community? - PROBE: Are any of these a yellow sweet potato? | | | |  |
| 15.f | |  | ◼ | [SHOW ALL S.P. PHOTOS]   - Which sweet potatoes are available in your community? | | | |  |
| 16.a | | 637e:  643d: | ◼ | I asked if you or (NAME) had “any dark green, leafy vegetables, such as nakati, sukumawiki, dodo, or gobe, or other dark green, leafy vegetable”.   - Name dark green, leafy vegetables based on these instructions. | | | |  |
| 16.b | |  | ◼ | Do you consider cabbage to be: (1) a “dark green, leafy vegetable” or (2) something else? [PROBE IF CABBAGE NOT LISTED BUT CONSIDERED DGLV HERE] | | | |  |
| 17.a | | 637f:  643e: | ◼ | I asked if you or (NAME) had “any other vegetables, such as tomato, bilinganya, green pepper, okra, mushroom, or other vegetable”.   - Name other vegetables based on these instructions. | | | |  |
| 17.b | |  | ◼ | When I asked if you or (NAME) had “any other vegetables”, did you consider potatoes? Why?  OTHER PROBES, IF NEEDED:   - Do you consider potatoes to be a vegetable? | | | |  |
| 17.c | |  | ◼ | When I asked if you or (NAME) had “any other vegetables”, did you consider onions? Why?  OTHER PROBES, IF NEEDED:   - Do you consider onionsto be a vegetable? | | | |  |
| 17.d | |  | ◼ | To clarify, are onions (1) more like a vegetable, or (2) more like a flavoring? | | | |  |
| 18. | | 637g:  637h:  643f:  643g: | ◼ | I asked if you or (NAME) had “ripe mango, ripe papaya, passionfruit, or binyaanya”. Then I asked if you or (NAME) had “any other fruits, such as banana, orange, pineapple, watermelon, owelo, or other fruit”.   - Name other fruits based on these instructions. | | | |  |
| 19.a | | 637i:  637j:  643h:  643i: | ◼ | I asked if you or (NAME) had “liver, offal, heart, kidney, lungs, pancreas, or sausage”. Then I asked if you or (NAME) had “any other meat, such as beef, goat, pig, rat, chicken, guinea fowl or bush meat”.   - Name other meats based on these instructions. | | | |  |
| 19.b | |  | ◼ | Do you think people will answer whether they or their child had meat honestly? Why? | | | |  |
| 20. | | 637r:  643q: | ◼ | I asked if you or (NAME) had “any sweet foods such as keki, cookies, biscuits, daddies, sweets, chocolate, or ice cream”.   - Name sweet foods based on these instructions. - IF NOT STATED: Do you consider honey to be a "sweet food"? | | | |  |
| 21.a | |  | ◼ | I asked if you or (NAME) had “packaged instant noodles”.   - What do you think I meant by “packaged instant noodles”?   OTHER PROBES, IF NEEDED:   - How do you prepare “packaged instant noodles”? | | | |  |
| 21.b | |  | ★ | IF ABLE TO ANSWER:   - Are “packaged instant noodles” available in your community? - If yes, which “packaged instant noodles” are available in your community? [PROBE: BRANDS] | | | |  |
| 22.a | |  | ★ | IF (NAME) ATE YOGURT OR BONGO YESTERDAY:   - When I asked, "how many times (NAME) ate solid, semi-solid, or soft foods yesterday", did you consider including yogurt eaten with a spoon? Why? | | | |  |
| 22.b | |  | ★ | IF (NAME) DID NOT EAT YOGURT OR BONGO YESTERDAY:   - When I asked, "how many times (NAME) ate solid, semi-solid, or soft foods yesterday", would you have considered including yogurt eaten with a spoon? Why? | | | |  |
| 22.c | |  | ◼ | Just to confirm, do you consider yogurt eaten with a spoon to be a "solid, semi-solid or soft food"? | | | |  |
| 23.a | |  | ◼ | I asked: “As part of your antenatal care during your pregnancy with (NAME), did a healthcare provider talk with you about which foods or how much food you should eat”. | | | |  |
| 23.b | | 418e | ★ | IF YES TO 418e: You said “yes”.   - How confident are you that a healthcare provider talked to you–while pregnant, before (NAME) was born–about which foods or how much food you should eat? OTHER PROBES, IF NEEDED: Do you remember it very well, or are you not certain? - What information did your healthcare provider give you about which foods or how much food you should eat while you were still pregnant with (NAME)? | | | |  |
| 23.c | | 418e | ★ | IF NO TO 418e: You said “no”.   - How confident are you that a healthcare provider did NOT talk to you–while pregnant, before (NAME) was born–about which foods or how much food you should eat? OTHER PROBES, IF NEEDED: Do you remember it very well, or are you not certain? - What information do you think a healthcare provider should give–during pregnancy–about which foods or how much food to eat? | | | |  |
| 24.a | |  | ◼ | I asked: “As part of your antenatal care during your pregnancy with (NAME), did a healthcare provider talk with you about breastfeeding”. | | | |  |
| 24.b | | 418f | ★ | IF YES TO 418f: You said “yes”.   - How confident are you that a healthcare provider talked to you–while pregnant, before (NAME) was born–about breastfeeding? OTHER PROBES, IF NEEDED: Do you remember it very well, or are you not certain? - What information did your healthcare provider give you about breastfeeding while you were still pregnant with (NAME) (before giving birth)? | | | |  |
| 24.c | | 418f | ★ | IF NO TO 418f: You said “no”.   - How confident are you that a healthcare provider did NOT talk to you–while pregnant, before (NAME) was born-about breastfeeding? OTHER PROBES, IF NEEDED: Do you remember it very well, or are you not certain? - What information do you think a healthcare provider should give-during pregnancy–about breastfeeding? | | | |  |
| 25.a | |  | ◼ | I asked: “In the first two days after (NAME’s) birth, where did (NAME) stay most of the time during the day and at night, ...”.   - What do you think I meant by “most of the time”?   OTHER PROBES, IF NEEDED:   - What does "most of the time" mean when I say [RELEVANT EXAMPLE]? | | | |  |
| 25.b | |  | ◼ | - In the first 2 days after delivery, how many times were you in a separate room from (NAME)? - IF AT ALL: How long were you in a separate room from (NAME) at each separation? | | | |  |
| 25.c | |  | ◼ | How do you remember where (NAME) stayed in the first 2 days after birth? | | | |  |
| 25.d | | 435: | ★ | IF DELIVERED IN FACILITY (435 code 21-36):   - When I asked about where (NAME) stayed in the first 2 days after delivery, were you thinking of (1) the time you were in the facility, (2) the time after you left the facility, or (3) both? | | | |  |
| 26.a | |  | ◼ | I asked: “During the first 2 days after (NAME)’s birth, did any healthcare provider ... Observe (NAME) breastfeeding”.   - How easy or difficult is it for you to remember if a healthcare provider observed you breastfeeding during the first 2 days after (NAME)’s birth?   OTHER PROBES, IF NEEDED:   - How confident are you about whether your healthcare provider observed (NAME) breastfeeding? Do you remember it very well, or are you not certain? | | | |  |
| 26.b | |  | ◼ | When I asked, “During the first 2 days after (NAME)’s birth, did any healthcare provider ... Observe (NAME) breastfeeding”:   - Who were you thinking about as someone that might have observed you breastfeeding (NAME)? | | | |  |
| 26.c | |  | ★ | IF HEALTHCARE PROVIDER NOT MENTIONED:  When I asked, “During the first 2 days after (NAME)’s birth, did any healthcare provider ... Observe (NAME) breastfeeding”:   - Would you have said yes or no if a healthcare provider had observed you breastfeeding (NAME)? | | | |  |
| 26.d | |  | ◼ | When I asked, “During the first 2 days after (NAME)’s birth, did any healthcare provider ... Observe (NAME) breastfeeding”:   - Would you have said yes or no if ONLY a family member had observed you breastfeeding (NAME)? Why? - Would you have said yes or no if a healthcare provider was present at the time you were breastfeeding (NAME) but was not observing you breastfeeding for the purpose of providing you support? Why? | | | |  |
| 27.a | |  | ◼ | - We need your help on the best way to ask this question for future surveys. - When asking whether a healthcare provider observed the mother breastfeeding in the first 2 days after birth, we only want the mother to say “yes” if a healthcare provider observed her breastfeeding. - If ONLY a family member or other non-healthcare worker observed the mother breastfeeding, we want the mother to answer “no”. - I will read to you two options for this question, and then ask which seems more likely to get an accurate response. - I can repeat them as often as necessary.   Option 1 is: During the first 2 days after (NAME)’s birth, did any healthcare provider “Observe that you are breastfeeding (NAME) correctly?”  Option 2 is: During the first 2 days after (NAME)'s birth, did any healthcare provider “Observe (NAME) breastfeeding?”   - Which option do you think is more likely to get an accurate response? Why? | | | |  |
| 28.a | |  | ◼ | I asked: “During the first 2 days after (NAME)’s birth, did any healthcare provider tell you where you could get help with breastfeeding?”.   - How easy or difficult is it for you to remember if a healthcare provider gave you information on where you could get help with breastfeeding?   OTHER PROBES, IF NEEDED:   - How confident are you about whether your healthcare provider gave you information on where you could get help with breastfeeding? Do you remember it very well, or are you not certain? | | | |  |
| 28.b | | 473f | ★ | IF YES TO 473f: You said "yes".   - What information did your healthcare provider give you about where to get help with breastfeeding? | | | |  |
| 28.c | | 473f | ★ | IF NO TO 473f: You said "no".   - What information do you think a healthcare provider should give about where to get help with breastfeeding? | | | |  |
| 29.a | |  | ◼ | How easy or difficult is it for you to remember if (NAME) was given anything other than breastmilk to eat or drink in the first 2 days after delivery? | | | |  |
| 29.b | | 483 | ★ | IF YES TO 483: You said (NAME) was given something other than breastmilk in the first 2 days after delivery.   - What was (NAME) given? | | | |  |
| 29.c | |  | ◼ | - Was (NAME) given anything to eat or drink as part of a religious ceremony or other ritual in the first 2 days after delivery? - If yes, what was (NAME) given? | | | |  |
| 29.d | |  | ◼ | - Was (NAME) given any medicines in the first 2 days after delivery? - If yes, what was (NAME) given? | | | |  |
| 29.e | |  | ◼ | Name (other) foods or liquids that children in your community may be given in the first 2 days after delivery. | | | |  |
| 29.f | |  | ◼ | Do you think most people would answer whether their child was given anything other than breastmilk to eat or drink in the first 2 days after delivery honestly? Why? | | | |  |
| 30.a | |  | ◼ | I asked: “In the last 3 months, has any healthcare provider or community health worker measured (NAME)'s weight, length or height, or around the upper arm”.   - Are any of these measurements performed on children in your community? | | | |  |
| 30.b | |  | ★ | IF YES:   - How are children measured? - Who typically measures children? PROBE: Anyone else? - Where do these measurements typically take place? | | | |  |
| 31.a | |  | ◼ | I asked, “In the last 6 months, did any healthcare provider or community health worker talk with you about how or what to feed (NAME)?”. | | | |  |
| 31.b | | 641 | ★ | IF YES TO 641: You said “yes”.   - What information did your healthcare provider give about what to feed (NAME)? - What information did your healthcare provider give about how to feed (NAME)? - Was it a healthcare provider, community health worker, other type of provider that talked with you? [IF OTHER PROVIDER]: What type of provider? | | | |  |
| 31.c | | 641 | ◼ | IF NO TO 641: You said “no”.   - What information do you think a healthcare provider or community health worker should give about what to feed a child? - What information do you think a healthcare provider or community health worker should give about how to feed a child? | | | |  |
| 32. | |  | ◼ | We have finished our questions.   - Do you have anything else you would like to add that we have not already talked about? | | | |  |
|  | |  | ◼ | Thank you for your time. I really appreciate your talking with me today.  TURN OFF RECORDER | | | |  |
| **End time:** | | | | | | | | |

**Nutrition Cognitive Interview Summary (VERSION 6)**

| Respondent Name |  |
| --- | --- |
| Cluster |  |
| Household number |  |
| Language |  |
| Date of interview |  |
| Interviewer(s) |  |
| Supervisor present (yes or no) |  |

- Describe any problems you had with administering a question(s) and any challenges observed on the behalf of the respondent being able to answer a question(s). Note any verbal or non-verbal cues that may be of interest (body language, hesitation, clarifying questions, requests repeat of questions, expresses uncertainty about their answer, provides uncodeable answer, among others). (USE BACK OF PAPER OR ADDITIONAL PAPER IF NEEDED)
- Describe any observations from the cognitive interview that is not captured from the recording. (USE BACK OF PAPER OR ADDITIONAL PAPER IF NEEDED)

**
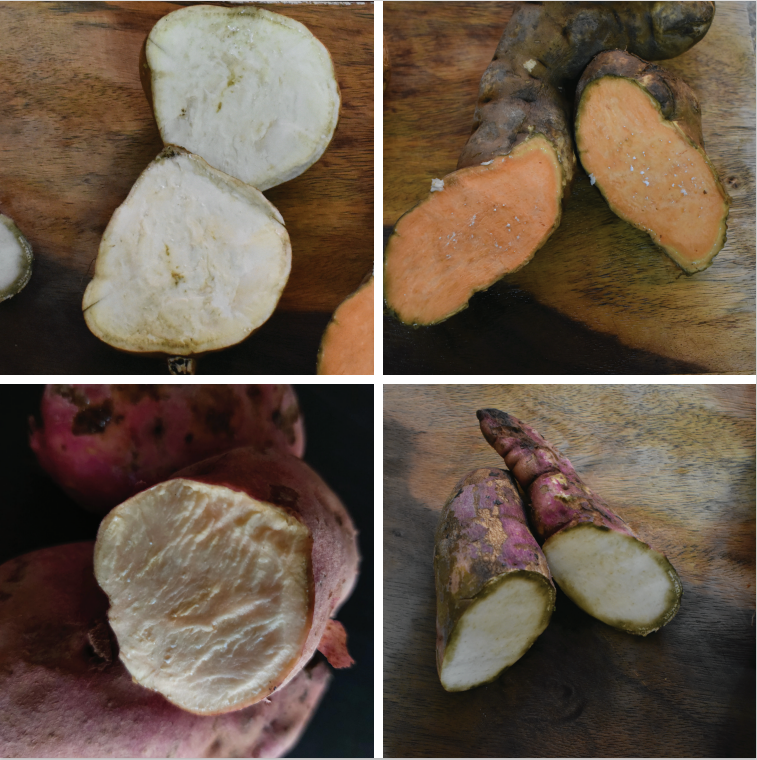
**

C

B

D\

A

**Supplementary Figure 1:** Respondents were randomly shown four pictures of sweet potatoes purchased from Kampala markets: A. Yellow unfortified sweet potato, B. Orange fortified sweet potato, C. Yellow fortified sweet potato, D. White sweet potato.
